# Inhibition of *Streptococcus pyogenes* biofilm by *Lactiplantibacillus plantarum* and *Lacticaseibacillus rhamnosus*

**DOI:** 10.1128/msphere.00430-24

**Published:** 2024-10-03

**Authors:** Alejandro Gómez-Mejia, Mariano Orlietti, Andrea Tarnutzer, Srikanth Mairpady Shambat, Annelies S. Zinkernagel

**Affiliations:** 1 Department of Infectious Diseases and Hospital Epidemiology, University Hospital Zurich, University Zurich, Zurich, Switzerland; University of Nebraska Medical Center College of Medicine, Omaha, Nebraska, USA

**Keywords:** *Streptococcus pyogenes*, biofilm, infectious diseases, antibacterial treatment, *Lactobacillus*

## Abstract

The human pathobiont *Streptococcus pyogenes* forms biofilms and causes infections, such as pharyngotonsillitis and necrotizing fasciitis. Bacterial biofilms are more resilient to antibiotic treatment, and new therapeutic strategies are needed to control biofilm-associated infections, such as recurrent pharyngotonsillitis. *Lactiplantibacillus plantarum* and *Lacticaseibacillus rhamnosus* are two bacterial commensals used for their probiotic properties. This study aimed to elucidate the anti-biofilm properties of *L. plantarum* and *L. rhamnosus* cell-free supernatants (LPSN and LRSN, respectively) on *S. pyogenes* biofilms grown *in vitro* in supplemented minimal medium. When planktonic or biofilm *S. pyogenes* were exposed to LPSN or LRSN*, S. pyogenes* survival was reduced significantly in a concentration-dependent manner, and the effect was more pronounced on preformed biofilms. Enzymatic digestion of LPSN and LRSN suggested that glycolipid compounds might cause the antimicrobial effect. In conclusion, this study indicates that *L. plantarum* and *L. rhamnosus* produce glycolipid bioactive compounds that reduce the viability of *S. pyogenes* in planktonic and biofilm cultures.

**IMPORTANCE:**

*Streptococcus pyogenes* infections are a significant concern for populations at risk, such as children and the elderly, as non-invasive conditions such as impetigo and strep throat can lead to severe invasive diseases such as necrotizing fasciitis. Despite its susceptibility to current antibiotics, the formation of biofilm by this pathogen decreases the efficacy of antibiotic treatment alone. The ability of commensal lactobacillus to kill *S. pyogenes* has been documented by previous studies using *in vitro* settings. The relevance of our study is in using a physiological setup and a more detailed understanding of the nature of the lactobacillus molecule affecting the viability of *S. pyogenes*. This additional knowledge will help for a better comprehension of the molecules’ characteristics and kinetics, which in turn will facilitate new avenues of research for its translation to new therapies.

## INTRODUCTION

*Streptococcus pyogenes* (group A *Streptococcus* [GAS]) is a human pathobiont that colonizes the throat and skin and may cause severe diseases (1) such as necrotizing fasciitis or streptococcal toxic shock syndrome with high mortality rates (2–4). *S. pyogenes* is also the most common bacterial cause of pharyngotonsillitis (5). Despite its full susceptibility to many antibiotics, including penicillin, antibiotic therapy alone is often not sufficient to eradicate *S. pyogenes* (6). After antibiotic treatment, up to 30% of pharyngotonsillitis remains symptomatic with *S. pyogenes* still detectable in cultures. One potential reason for the treatment failure in pharyngotonsillitis is the development of biofilms (7). Biofilm-forming microorganisms are responsible for a large number of difficult-to-treat infections worldwide (8, 9). Bacteria in biofilms are difficult to eradicate, due to insufficient antibiotic penetration into biofilms and the presence of metabolically inactive bacteria inside biofilms (10, 11). Biofilm formation is recognized as a common strategy for both Gram-positive and Gram-negative bacteria to persist and survive host defenses and antimicrobials. Biofilms are characterized by sessile aggregates encased in a self-produced matrix of extracellular polymeric substances and are found on biological and non-biological surfaces (12). It has been suggested that 99% of the world’s bacteria exist in a biofilm state and that biofilm bacteria vary drastically in their physiology, growth rate, and gene expression as compared to their planktonic counterparts (13). At least two-thirds of bacterial infections are estimated to be biofilm-related (14). Biofilms provide an enhanced structural defense against biological, physical, and chemical stressors (15) and thus biofilm-associated bacteria are difficult to eradicate. As antibiotics are often not sufficient to control biofilm-associated infections, mechanical removal by surgery may be required but is not always possible. Additional strategies to prevent and treat biofilms, such as the use of commensal bacteria aiming to outcompete and inhibit the growth of human pathogens (16–19), recently gained relevance. In most of these scenarios, prior colonization by commensal bacteria has been shown as the main effector due to the depletion of available nutrients for the competing pathogens or the established presence of antibacterial molecules (16, 20–22).

Some of the most commonly used probiotics are members of the former genus *Lactobacillus*, recently redistributed into 23 new genera (23). They belong to the normal mucosal microbiota of humans and animals and have rarely been associated with disease (24). They are found predominantly colonizing mucosal tissue, including the oropharynx (20, 24, 25). The former *Lactobacillus* spp. exhibit remarkable antimicrobial activity against pathogenic bacteria thanks to the production of bacteriocins, reactive oxygen species, biosurfactants, and exopolysaccharides with anti-biofilm activity (26–28). *Lactiplantibacillus plantarum* (LP) and *Lacticaseibacillus rhamnosus* (LR) belong to the human microbiota (29, 30) and are present in food as probiotics. Previous studies have demonstrated their anti-pathogen properties against *Streptococcus mutans*, *Candida* spp., *Pseudomonas aeruginosa*, *Staphylococcus aureus*, *Salmonella* sp., and uropathogenic *Escherichia coli* (26, 31–33). Since recurring *S. pyogenes* pharyngotonsillitis is such a large problem and the probiotics LP and LR are readily available and would represent an alternative for reducing prolonged antibiotic prescription, this study aimed to provide insights into the antibacterial mechanisms of *L. plantarum* and *L. rhamnosus* cell-free supernatants on *S. pyogenes* biofilms.

## RESULTS

### *S. pyogenes* grows and forms biofilms in defined cell culture media supplemented with bacterial medium

**Fig 1:**
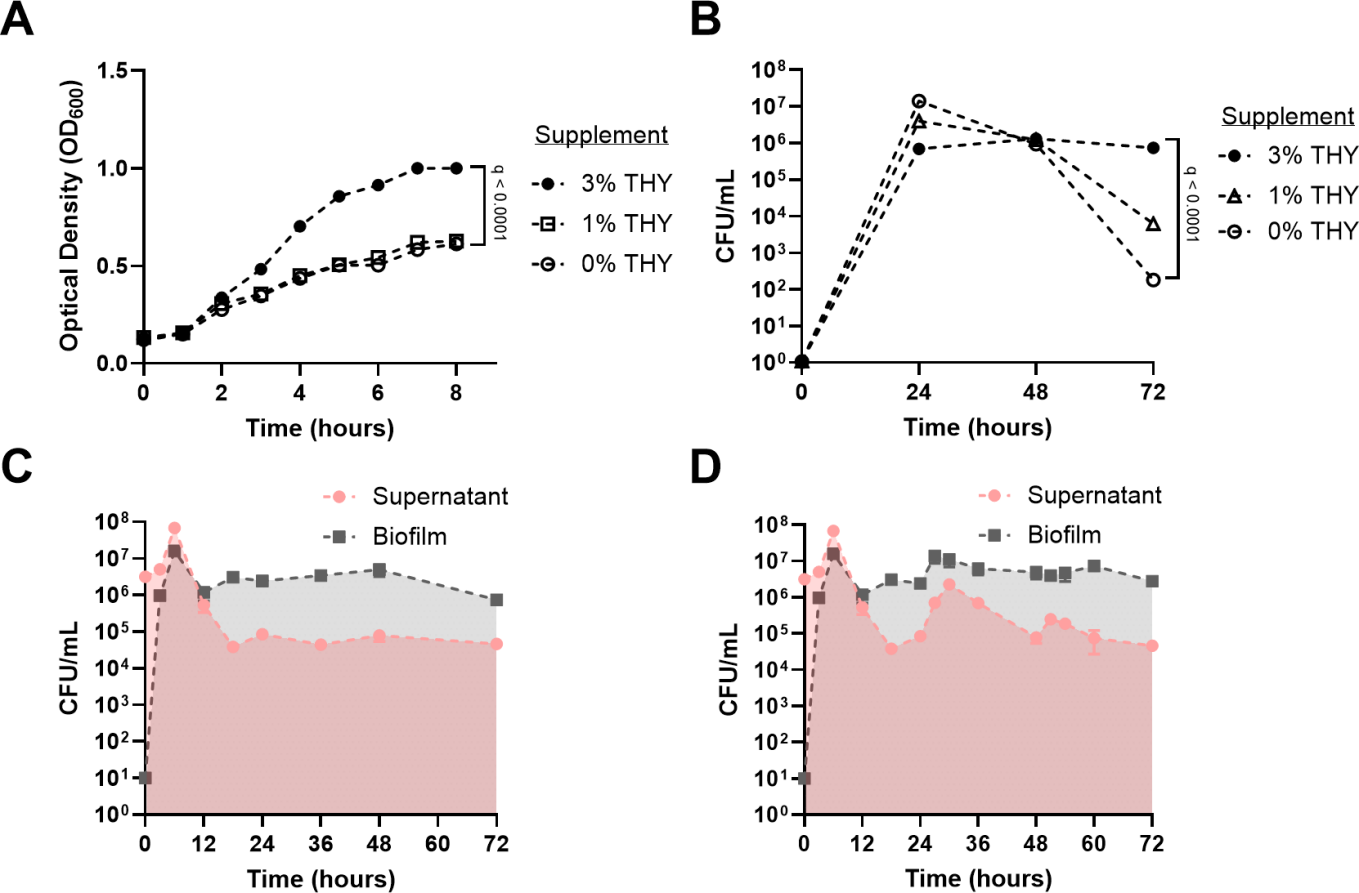
*S*. *pyogenes* growth and biofilm formation in RPMI supplemented with defined concentrations of THY. (**A**) Planktonic *S. pyogenes* growth assessed by optical density (OD_600_) in liquid RPMI supplemented with defined concentrations of THY. (**B**) Planktonic *S. pyogenes* survival in RPMI supplemented with different concentrations of THY. (**C**) *S. pyogenes* biofilm formation and viability in RPMI supplemented with 3% THY without medium exchange, determined by CFU/mL. (**D**) *S. pyogenes* biofilm formation and viability in RPMI supplemented with 3% THY and medium exchange every 24 hours, determined by CFU/mL. Data are shown as mean ± SD of three independent replicates (*n* = 3). The statistical test used was a non-linear regression model followed by a one-way ANOVA of the growth rate (**A**) or the maximum population (**B**) with Benjamini, Krieger, and Yekutieli multiple comparison correction for false discovery rate (**A and B**).

In order to mimic physiological nutrient conditions as found in biofilms in the host, *S. pyogenes* 5448 strain was grown in the defined cell culture medium RPMI1640 (RPMI) with and without bacterial medium (Todd-Hewitt yeast [THY]) supplementation. *S. pyogenes* 5448 grew in RPMI if supplemented with 1% THY; however, a minimum of 3% THY was needed to maintain cell viability beyond 72 hours (Fig. 1A and B). Furthermore, 3% THY was also the minimum supplement necessary for stable bacterial survival for 72 hours *in vitro* in biofilms without renewal of the growth medium (batch condition) (Fig. 1C). Biofilm formation was observed after 3 hours of growth as confirmed by the recovery of bacteria after thorough washing followed by sonication (Fig. 1C and D). Additionally, exchanging the growth medium with fresh medium every 24 hours significantly increased *S. pyogenes* 5448 biofilm survival at the 72-hour time point (Fig. 1D) (viable *S. pyogenes* counts above 10^6^ colony-forming units [CFUs]/mL) as compared to batch condition (viable *S. pyogenes* counts below 10^6^ CFU/mL). The formation of *S. pyogenes* 5448 biofilm in RPMI supplemented with 3% THY was further confirmed by crystal violet staining (Fig. S1).

### Antibacterial activity of *L. plantarum* and *L. rhamnosus* is medium dependent

**Fig 2:**
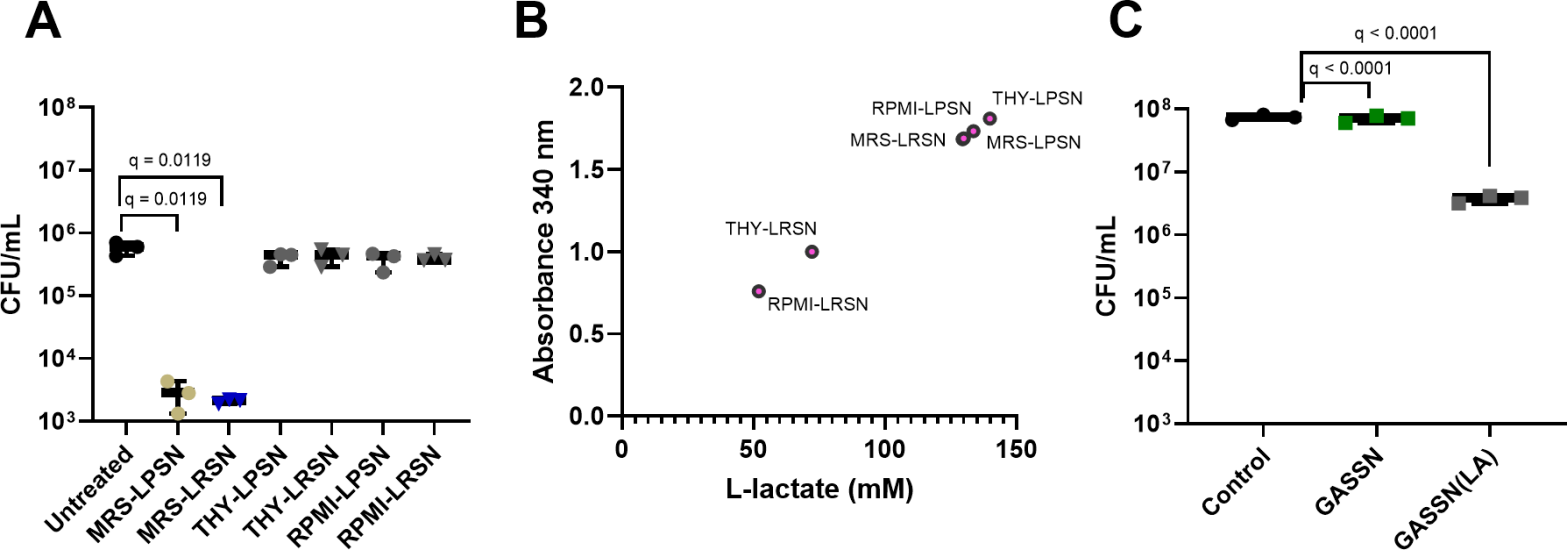
Lactic acid concentration and biofilm treatment with 5% LPSN or LRSN grown in different growth media. (**A**) *S. pyogenes* viable colonies recovered from 72-hour-old biofilms (media exchanged every 24 hours) after treatment with 5% LPSN or LRSN grown in MRS, THY, or RPMI for 48 hours (**B**) fitted values of the experimentally determined concentration of L-lactate (mM) from LPSN and LRSN after growth in RPMI, THY, and MRS for 24 hours (red circles). (**C**) *S. pyogenes* viable colonies recovered from 72-hour-old biofilms (media exchanged every 24 hours) after treatment with GASSN adjusted with 140 mM lactic acid for 48 hours. “control,” untreated; “GASSN(LA),” biofilms treated with GASSN with the addition of 140 mM lactic acid. For panels A and C, biofilms were allowed to form for 24 hours prior to the treatment. Data are shown as mean ± SD of three independent replicates (*n* = 3). Statistical significance is indicated as *q*-values. The statistical tests used were an ordinary one-way ANOVA with Benjamini, Krieger, and Yekutieli multiple comparison correction for false discovery rate (**A**) and a Kruskal-Wallis with Benjamini, Krieger, and Yekutieli multiple comparison correction for false discovery rate (**B**).

We next assessed the effect of rich bacterial media (THY and De Man Rogosa and Sharpe [MRS]) or physiological media (RPMI) on the anti-bacterial capacity of both lactobacilli toward *S. pyogenes* 5448 biofilms. Lactobacilli cell-free supernatants prepared using MRS media significantly reduced the viability of 72-hour-old *S. pyogenes* 5448 biofilms (approximately 2.5-log reduction) (Fig. 2A). In contrast, we observed that cell-free supernatants prepared using RPMI or THY did not affect the viability of similar *S. pyogenes* 5448 biofilms under the same treatment conditions (Fig. 2A). Next, we determined the effect on the production of lactic acid in the different media as the role of lactic acid, a metabolite produced by *Lactobacillus* spp., is known for its antibacterial properties. The L-lactate content was assessed in all supernatants with the highest content found in MRS supernatants and the lowest in RPMI supernatants (Fig. 2B). It is also important to note that the content of lactic acid from growth in MRS was similar for both strains of lactobacilli but differed when grown in RPMI or THY, with the highest concentration of lactic acid found in LP supernatants (Fig. 2B). Adding lactic acid to *S. pyogenes* 5448 cell-free spent medium adjusted to pH 4.5 (GASSN-LA) to a concentration comparable to the one present in *L. plantarum* and *L. rhamnosus* cell-free supernatants (LPSN and LRSN) in MRS (140 mM) showed a significant reduction of viable *S. pyogenes* 5448 recovered from 72-hour-old biofilms, albeit lower than with MRS-derived LPSN or LRSN (Fig. 2C).

### *L. plantarum* and *L. rhamnosus* cell-free supernatants reduce *S. pyogenes* survival in liquid cultures and biofilm in a concentration-dependent manner

**Fig 3:**
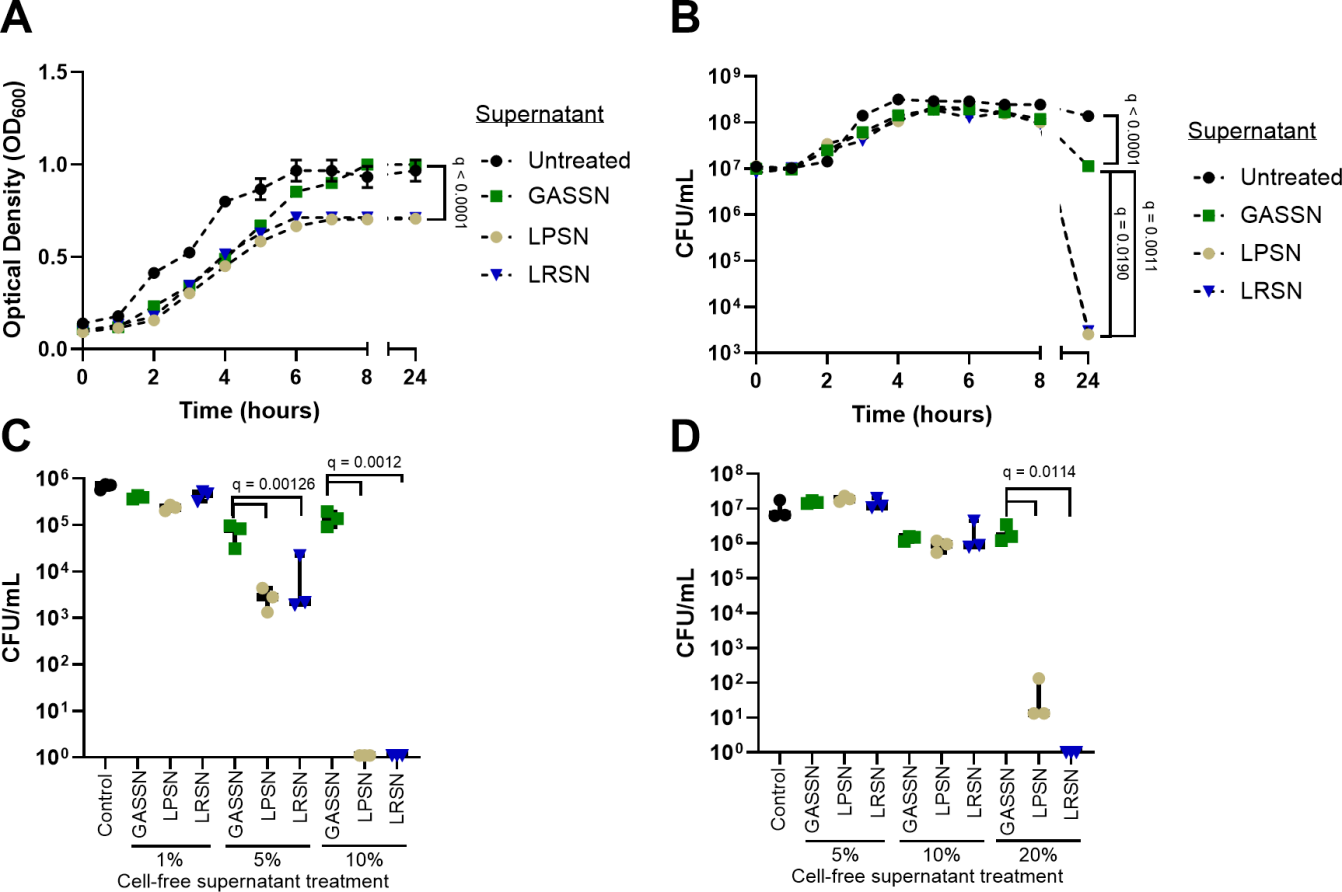
Inhibitory effect of LP and LR-cell-free supernatants on *S. pyogenes* growth in liquid cultures and 72-hour biofilms. Determination of *S. pyogenes* growth by optical density (OD_600_) (**A**) or as CFU/mL (**B**) after the addition of 5%(vol/vol) of LPSN (LP) or LRSN (LR) in comparison to GASSN (*S. pyogenes* spent medium adjusted to pH 4.5). (**C**) Viable *S. pyogenes* CFU/mL counts recovered from 72-hour biofilms without media exchange after 48 hours of treatment. (**D**) Viable *S. pyogenes* CFU/mL counts recovered from 72-hour biofilms with media exchange (every 24 hours) after 48 hours of treatment. For panels C and D, biofilms were allowed to form for 24 hours prior to treatment. Control, untreated. Data are shown as mean ± SD of three independent replicates (*n* = 3). Statistical significance is indicated as *q*-values. The statistical tests used were a non-linear regression model followed by a one-way ANOVA analysis of the growth rate (**A**) or the population at peak (**B**) and an ordinary one-way ANOVA (**C and D**). All cases were analyzed with correction for false discovery with Benjamini, Krieger, and Yekutieli multiple comparison.

The presence of 5% *L*. *plantarum* cell-free MRS supernatant and *L. rhamnosus* cell-free supernatant in supplemented RPMI cultures led to a significant reduction in *S. pyogenes* 5448 bacterial growth (Fig. 3A and B). After 24 hours of incubation, a significant reduction in *S. pyogenes* viable cells was observed when treated with either LPSN or LRSN compared to the control group treated with *S. pyogenes* cell-free spent medium with pH adjusted to 4.5 (GASSN) or just MRS medium (Fig. 3A and B). The observed inhibitory effect with 5% LPSN and LRSN was also evaluated in three additional *S. pyogenes* strains, including an additional M1 strain, one M28, and one M90 strain (Fig. S2). As an additional control, we evaluated the effect of 5% LPSN and LRSN on the growth and viability of an *S. aureus* USA300 JE2 strain compared to a control group treated with *S. aureus* cell-free spent medium with pH adjusted to 4.5 (SASN). Here, no growth effect was observed from the treatments (Fig. S4A).

In order to evaluate the anti-*S. pyogenes* biofilm activity of LP and LR, different concentrations of MRS LPSN or LRSN (1%, 5%, 10%, or 20% [vol/vol]) were added to 24-hour-old *S. pyogenes* biofilms. After 48 hours of biofilm treatment (72 hours of experiment) without medium exchange, a significant reduction of viable *S. pyogenes* was observed with 10% LPSN or LRSN compared to the GASSN control (Fig. 3C). However, when the growth medium was exchanged every 24 hours, a treatment with 20% (vol/vol) LPSN or LRSN was required to achieve a significant reduction of viable cells (Fig. 3D). Similarly, 20% LPSN or LRSN treatment significantly reduced biofilms formed by additional *S. pyogenes* strains M28, M90, and an additional M1 strain (Fig. S3). We also assessed the effect of SASN at 20% on the biofilm viability of *S. pyogenes* and observed no significant differences indicating no inhibition (Fig. S4B).

### Efficiency of the antibacterial action of LP and LR cell-free supernatants is dependent on the growth state of *S. pyogenes*

**Fig 4:**
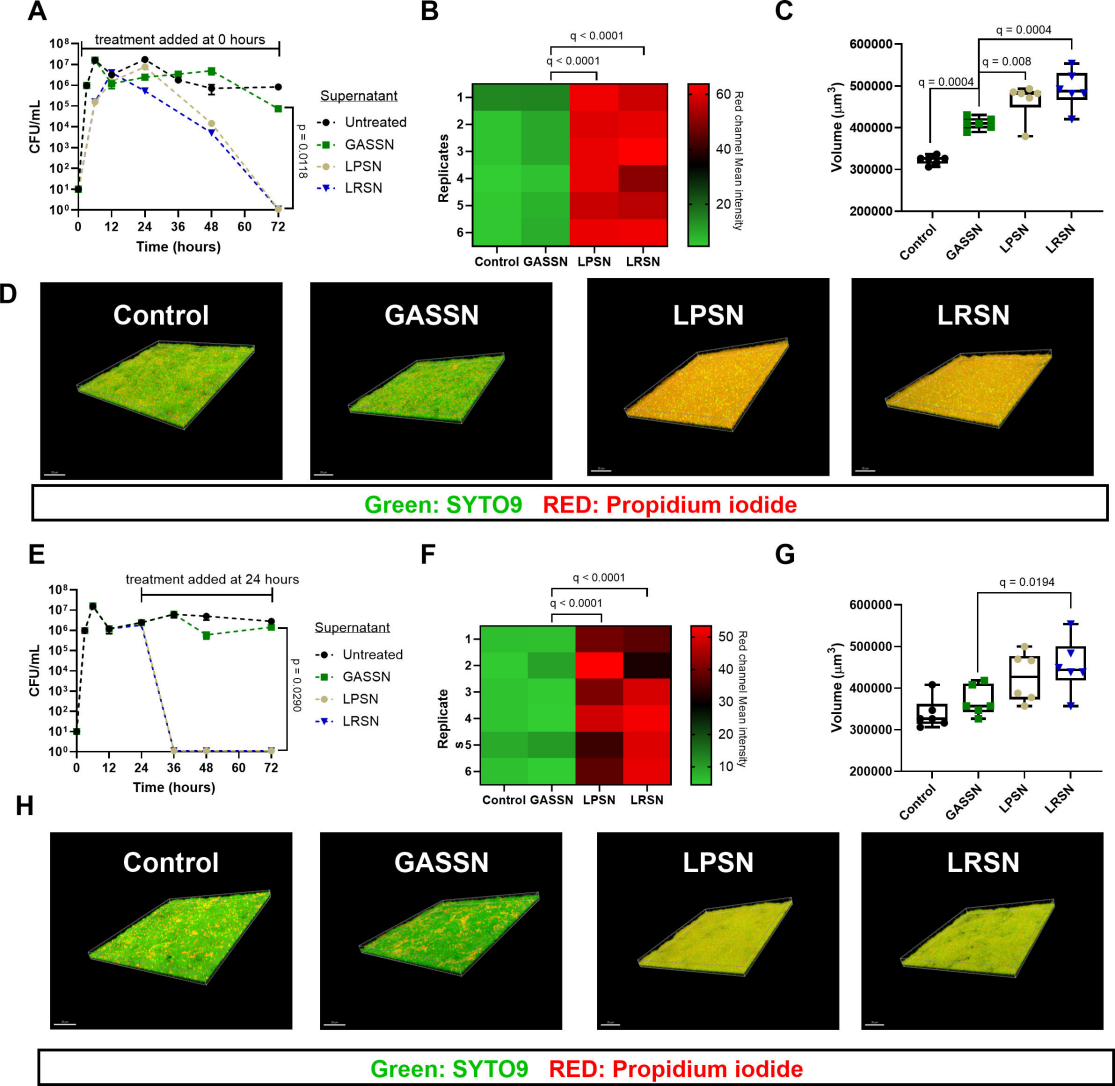
Effect of *L. plantarum* and *L. rhamnosus* cell-free supernatants on *S. pyogenes* biofilms. (**A–D**) 72 hours of *S. pyogenes* biofilm formation and viability when 0-hour-old biofilms were treated with 20% LPSN or LRSN for 72 hours. (**E–H**) 72 hours of *S. pyogenes* biofilm formation and viability when 24-hour-old biofilms were treated with 20% LPSN or LRSN for 48 hours. Media were exchanged every 24 hours (**A–H**). (**A and E**) Viable *S. pyogenes* log_10_ CFU/mL counts recovered at 72 hours of experiment. Data are shown as mean ± SD of three independent replicates (*n* = 3). (**B and F**) *S. pyogenes* biofilm mean intensity of red (propidium iodide) fluorescence channel after measurement in confocal microscopy at 72 hours of experiment. (**C and G**) *S. pyogenes* biofilm volume in µm^3^ measured with the image scale values at 72 hours of experiment. Data are shown as mean ± SD of two representative slide spots of three independent biological replicates (*n* = 6). (**D and H**) Representative 3D merged channels of *S. pyogenes* biofilm models at 72-hour time point from each treatment group. Scale 30 µm. Statistical significance is indicated as *P* or *q*-values. The statistical test used was an ordinary one-way ANOVA (**A and E**) or a mixed-effect analysis (**B, C, F, and G**) with Benjamini, Krieger, and Yekutieli multiple comparison correction for false discovery rate.

Addition of 20% LPSN or LRSN to planktonic *S. pyogenes* 5448 cultures before biofilm development (hour 0) did not prevent biofilm formation (Fig. 4A). However, a decrease in bacterial survival was observed after 24 hours of treatment with an estimate of 48–72 hours of treatment required to reduce the number of viable cells to undetectable levels (Fig. 4A). Corroborating these findings, the LIVE/DEAD BacLight staining imaged by confocal microscope showed a significant increase in damaged or dead cells inside the biofilm reported as the mean intensity of the fluorescence emitted by propidium iodide in both LPSN and LRSN treated groups (Fig. 4B). Of interest, even though no viable cells were recovered after 72 hours of treatment, a biofilm volume analysis of the confocal images showed a significantly larger biofilm volume after treatment with either 20% of LPSN or LRSN (Fig. 4C). When evaluating the effect on 24-hour-old biofilms, the addition of 20% LPSN or LRSN led to rapid cell death at 36 hours (12 hours after treatment started) (Fig. 4E). Microscopic analysis showed a significant increase in the fluorescence signal measured from the staining with propidium iodide after 48 hours of treatment with 20% LPSN or LRSN (Fig. 4F). A significant increase in biofilm volume was observed only after treatment with 20% LRSN (Fig. 4G). The 3D figure of scanned biofilms with all colors shows the damage caused by the treatments as the intensity of the propidium iodide increases (Fig. 4D and H). 2D representations of all samples can be found in Fig. S5 and S6.

### A glycolipidic compound drives the antibacterial capacity of *L. plantarum* and *L. rhamnosus* cell-free supernatants toward *S. pyogenes* biofilms

**Fig 5:**
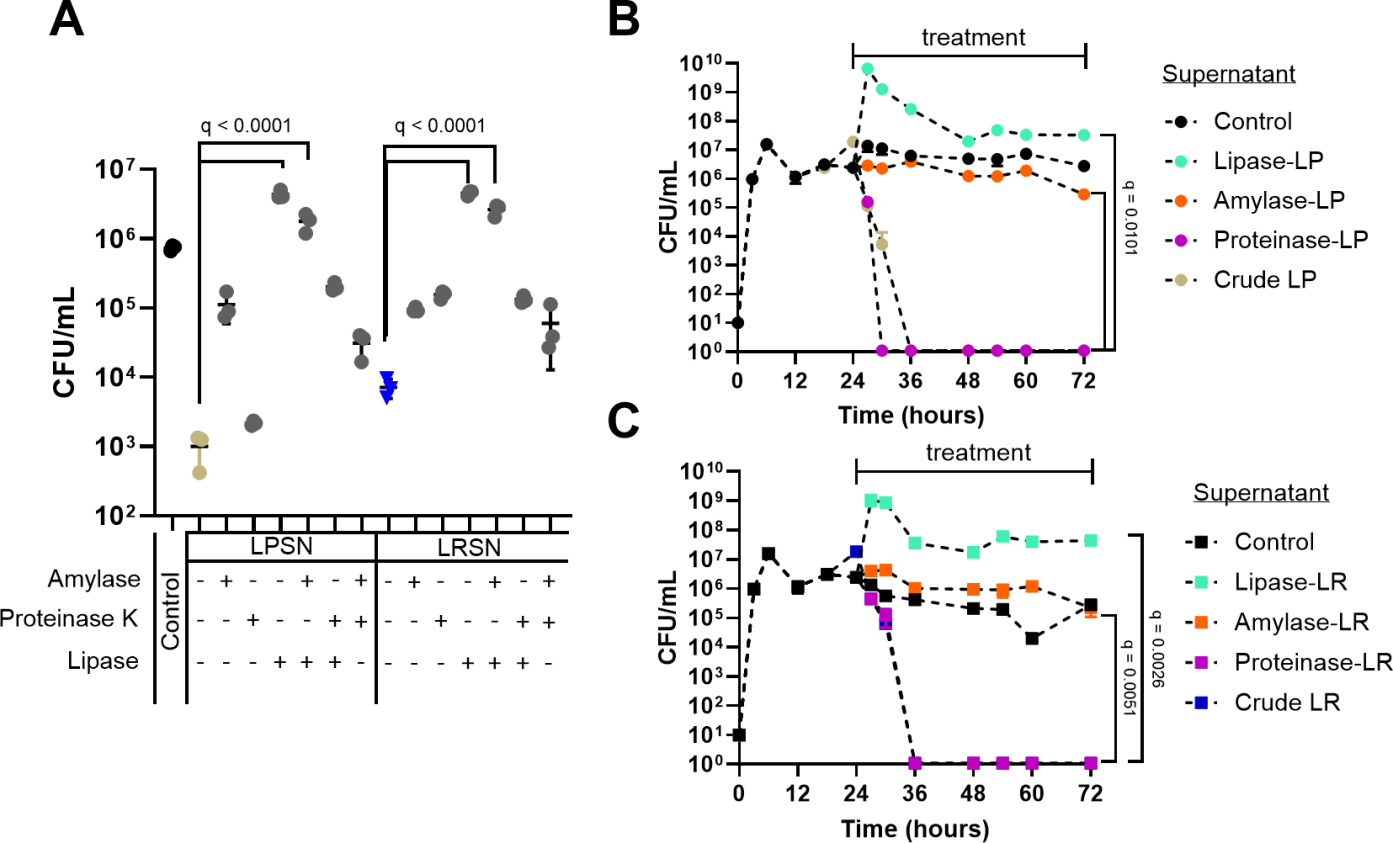
*S. pyogenes* biofilm-inhibitory effect of single- and double-enzymatic treatment of *L. plantarum* and *L. rhamnosus* supernatants. Twenty-four-hour-old *S. pyogenes* 5448 biofilms grown in RPMI supplemented with THY were treated with different digestions (proteinase K, α-amylase, or lipase alone or in different combinations) of 20% MRS LPSN or LRSN for 48 hours (with medium exchange every 24 hours). (**A**) Number of viable bacteria recovered from biofilms at the 72- hour time point. (**B and C**) Kinetics of *S. pyogenes* 5448 viability in the biofilms under treatment with digested 20% LPSN (**B**) or 20% LRSN (**C**). GASSN at a concentration of 20% was used as a control. Data are shown as mean ± SD of three independent replicates (*n* = 3). Statistical significance is indicated as *q*-values. The statistical tests used were an ordinary one-way ANOVA (**A**) or a mixed-effect analysis (**B and C**) with Benjamini, Krieger, and Yekutieli multiple comparison correction for false discovery rate.

In order to categorize potential extracellular metabolites responsible for the antibacterial activity of LPSN and LRSN against *S. pyogenes* biofilms, single and double enzymatic treatments of LPSN and LRSN with proteinase K, α-amylase, and lipase were performed. The highest loss of the inhibitory capacity was observed upon lipase treatment for both LPSN and LRSN supernatants (Fig. 5A). A significant reduction of the inhibitory capacity was also observed upon treatment with a combination of lipase and α-amylase for both supernatants (Fig. 5A). No significant changes were observed upon treatment with proteinase K (Fig. 5A). Kinetic experiments confirmed the loss of antibacterial activity of MRS, LPSN, and LRSN treated with either lipase or amylase (Fig. 5B and C). Control experiments were performed to evaluate the stability of the active molecule in the LPSN and LRSN and to evaluate the effect of enzymatic activity from proteinase K, α-amylase, and lipase on the viability of the biofilm directly. A 1-log decrease was observed in the antibacterial activity of both LPSN and LRSN when exposed to 95°C heat for 3 minutes, a step needed to inactivate the enzymes used for the enzymatic digestions, which indicates that the molecule is thermostable (Fig. S7).

Fourier transmission infrared spectroscopy (FTIR) was performed on LPSN and LRSN in order to identify functional groups of organic compounds based on the characteristic infrared absorption peaks of target chemical groups. When LPSN and LRSN grown in MRS were analyzed, the main difference was observed at 3,200–3,600 cm^−1^ spectra, corresponding to OH and ND groups. While in pure MRS, the detected signals were weak, a medium OH and a weak NH signal were detected for MRS LPSN, and both medium OH and NH signals were detected in MRS LRSN (Table 1).

**TABLE 1:** FTIR spectrum ranges and functional groups detected in *L. plantarum* and *L. rhamnosus* cell-free supernatants grown in MRS (MRS LPSN and MRS LRSN) compared to fresh growth medium (MRS control)

Spectral range (cm^−1^)	Functional groups detected		
	MRS control	MRS LPSN	MRS LRSN
Below 1,000	CN strong stretching	CN weak stretching	CN weak stretching
1,000–1,300	C-O strong stretching; OH strong deformation	C-O strong stretching; OH weak deformation	C-O strong stretching; OH strong deformation
1,400–1,460	CH_2_ and CH_3_ medium asymmetric deformation	CH_2_ and CH_3_ weak antisymmetric deformation	CH_2_ and CH_3_ medium deformation
1,490–1,540	NH strong deformation	NH strong stretching	NH strong combination
1,675–1,725	C = O strong stretching	C = O strong stretching	C = O strong stretching
3,200–3,600	OH and NH weak stretching	OH medium; and NH weak stretching	OH and NH medium stretching

## DISCUSSION

In this study, we showed that four different *S. pyogenes* strains with three different *emm* types (M1, M28, and M90 [34, 35]) formed biofilms *in vitro* in a well-defined chemical medium when supplemented with THY. The selection for the *S. pyogenes* strains and *emm* types used in this study was based on their epidemiological importance with the M1 type being reported as the most prevalent in *S. pyogenes*-derived invasive disease, while M28 and M90 *emm* types have also been reported in cases of invasive *S. pyogenes* (34, 35). We further showed that the treatment of *S. pyogenes* biofilms with *L. plantarum* and *L. rhamnosus* supernatants reduced the number of viable *S. pyogenes* in the biofilm significantly. This anti-biofilm effect was more pronounced when the cell-free supernatants were added to biofilms as compared to planktonic bacteria and was observed to be consistent among the different *S. pyogenes* strains evaluated. The anti-*S. pyogenes* biofilm property of LP and LR cell-free supernatants is possibly bound to a variety of bioactive compounds of the lipids or glycolipids class produced by these two commensal species.

*S. pyogenes* is known to be a well-adapted human pathogen capable of colonizing mucosal surfaces such as the oropharynx and the skin, causing infections such as tonsillitis, impetigo, and necrotizing fasciitis (3, 6, 36). An overall antibiotic failure rate of 20%–40% has been reported for *S. pyogenes* (37), although *S. pyogenes* antibiotic susceptibility studies have reported no resistance to penicillin so far, and the mechanism for this resistance remains elusive (38). In addition, antibiotic resistance to macrolides, tetracycline, and fluoroquinolones has been reported (39). Thus, due to evolving antibiotic resistance as well as the intrinsic antibiotic tolerance of bacteria in biofilms, new infection control approaches are needed. Biofilm-related infections represent a challenge in terms of infection control (7, 40, 41). Biofilm-like *S. pyogenes* communities have been observed in tonsillar reticulated crypts, suggesting a role for biofilm in asymptomatic *S. pyogenes* carriage (3, 42). Biofilm formation has also been reported in impetigo lesions caused by *S. pyogenes* (4) and in throat-colonizing isolates (7, 43).

Cell culture media are well-defined minimal media used to culture eukaryotic cells but not usually bacteria, which are grown in extremely rich and not well-defined media. Previous studies have shown that the mucosal pathobionts *Candida albicans*, *Staphylococcus aureus,* and *Pseudomonas aeruginosa* form *in vitro* biofilms in the tissue culture medium RPMI (44–46). Aiming to use a physiological medium that mimics the composition of human fluids (47), we assessed *S. pyogenes* growth, biofilm formation, and survival in RPMI. To our knowledge, the *in vitro* growth and biofilm formation capacity of *S. pyogenes* in supplemented RPMI have not been investigated before. Supplementation with THY does not mimic physiological conditions but was necessary to allow for bacterial growth and survival in liquid cultures as well as biofilm formation for the duration of the experiments performed in this study (72 hours). The decreased biofilm viability in RPMI alone or supplemented with less than 3% THY might be due to a lack of nutrients.

The antibacterial effect of probiotic bacteria on important human pathogens, such as carbapenem-resistant *Enterobacteriaceae* (48), *Salmonella enterica*, *Staphylococcus aureus,* and *Candida albicans* (49, 50), was shown in previous studies (26). Probiotics include commensal bacteria that display several antibacterial properties, including the former genus *Lactobacillus* (51). These commensals can also be found co-inhabiting the same human niche as other pathobionts such as *S. aureus* and *S. pyogenes* (20, 24, 25). These cases lead to interactions among the different bacterial communities and competition for nutrients and space in which probiotic bacteria have been shown to be important due to their ability to limit the growth of pathobionts (16, 20–22). In the case of *Lactobacillus* antibacterial activity, a significant effect is attributed to the production of lactic acid as a growth inhibitory substance (52–56). Here, we showed that the loss of both *S. pyogenes* planktonic and biofilm viability was reduced more by treatment with LP or LR supernatants than *S. pyogenes*-spent media adjusted with lactic acid, suggesting that lactic acid was not the main cause for the antibacterial activity. Additionally, we observed that the presence of LPSN or LRSN during the initial stages of biofilm formation did not prevent cell attachment. Nevertheless, the treated biofilms were not viable anymore after 72 hours of exposure to LPSN or LRSN. This could indicate that planktonic *S. pyogenes* are less susceptible to treatment with LPSN and LRSN than those already embedded in a biofilm. Similar findings were described before for other *Streptococcus* spp. (57, 58), suggesting that environmental low pH can enhance biofilm formation as an adaptive response. In addition, when pre-formed biofilms (24 hours of growth) were treated with LPSN or LRSN, a drastic and almost instantaneous decrease in biofilm viability was confirmed by a significant reduction in CFU numbers within 6–12 hours after treatment. Hence, by treating *S. pyogenes* in its planktonic state and biofilm state, we observed a higher susceptibility of pre-formed biofilm, which was not evaluated in similar studies conducted previously. In parallel to the inhibitory effect of LP and LR on *S. pyogenes*, we also evaluated the inhibitory effect on *S. aureus*, another human commensal. Our results indicated no inhibitory activity from 5% LPSN or LRSN. Previous studies have reported mixed results on the antimicrobial effect on the growth of *S. aureus* by LP and LR. Inhibition of *S. aureus* was shown by using live LR bacterial cells and no effect when spent media from LR was used (59), the latter being consistent with our findings. On the other hand, a significant inhibition of *S. aureus* using both live cells and cell-free supernatant has been reported for both LP and LR (22, 60). The discrepancies between the different results might come from the different conditions and *S. aureus* strains used for the assays, which indicate that specific conditions such as the medium in which the lactobacilli is grown and the *S. aureus* strain evaluated might play a role and could explain our findings.

Consistent with our findings, a significant antagonistic effect of *L. plantarum* on *S. pyogenes* biofilms in the absence of significant acidification or cell-cell contact has been reported (61). Humphreys and McBain (61) proposed that the antagonistic effect on *S. pyogenes* might be linked to plantaricin, a peptide produced by *L. plantarum*. We, however, demonstrated that treatment of *L. plantarum* cell-free supernatant with proteinase K does not affect its antibacterial effect on *S. pyogenes* biofilms. Danilova et al. (62) assessed the effect of proteinase K on *L. plantarum* cell-free supernatants and, consistent with our observations, only non-peptide components were responsible for the antimicrobial activity against *E. coli*, *P. aeruginosa, S. aureus,* and *S. pyogenes*. *L. rhamnosus* cell-free supernatants were previously shown to inhibit cell-surface components mediating eukaryotic cell invasion by *S. pyogenes in vitro* (21). Furthermore, the effect of *L. rhamnosus* on the adherence of *S. pyogenes* to eukaryotic cells was studied, suggesting that effector molecules released by *L. rhamnosus* and other former *Lactobacillus* strains attenuate the production of virulence factors involved in *S. pyogenes* colonization; nevertheless, no specific secreted metabolites were identified (63). Our data suggest that lipidic or glycolipidic compounds may be involved in the observed antibacterial effect since only the treatment with lipase inhibited the anti-biofilm properties of both cell-free supernatants. Although no specific lipidic compounds have been directly correlated with the antimicrobial activity against *S. pyogenes*, recent studies (64) have reported the presence of lipidic biosurfactants with predominantly elaidic and palmitic fatty acids in *L. plantarum* cell-free supernatant. Our data suggest that the anti-biofilm potential of both LP and LR is not only dependent on the lactic acid production or acidic pH.

Fourier-transform infrared spectroscopy has been used before to characterize the functional groups associated with biosurfactants present in supernatants derived from different species of the former genus *Lactobacillus*. Based on our results, a more intense band detected at 3,200–3,600 cm^−1^ suggested the presence of carbohydrates or glycoproteins in all LP and LR supernatants when compared to the medium alone. This may be attributed to lipidic ester bonds present in biosurfactants secreted by *L. plantarum* and *L. rhamnosus*. Furthermore, it was reported that *Lactobacillus delbrueckii* and *Lactobacillus helveticus* produce glycolipidic biosurfactants (65, 66). Other lactic acid bacteria produce glycoproteic biosurfactants containing glucose, mannose, fructose, and rhamnose residues (67, 68). Based on the enzymatic treatment and FTIR analysis of LPSN and LRSN, we suggest that both species produce glycolipidic biosurfactants and that these biosurfactants are responsible for the antibacterial effect on *S. pyogenes*. However, this remains a hypothetical suggestion since the purification of the molecule was not achieved in this study and presents an important limitation for the scope of this study.

Our study provides new information on the mechanism of biofilm inhibition by *L. plantarum and L. rhamnosus*. There are, however, still certain limitations that need to be acknowledged as they provide the necessary foundation for the continuation of similar studies. The fact that the antibacterial effect was only observed when *S. pyogenes* biofilms were treated with LPSN or LRSN derived from cultures using MRS medium suggests that the production of the antibacterial molecule by LP and LN is medium dependent. As the next steps, it would be of interest to delve deeper into the antimicrobial potential of LP and LR in more complex setups such as *ex vivo* tissues or organoid models.

Overall, in this study, we showed that cell-free supernatants derived from *L. plantarum* and *L. rhamnosus* MRS liquid cultures exhibited a significant anti-bacterial potential against *S. pyogenes* in planktonic as well as biofilms *in vitro*. Although further studies are required, the anti-bacterial agent is suspected to be a lipidic or glycolipidic compound. This confirms that both *L. plantarum* and *L. rhamnosus* produce and secrete bioactive compounds that could have an important therapeutic value, which could potentially be used in the future to reduce *S. pyogenes* biofilms in conjunction with antibiotics.

## MATERIALS AND METHODS

### Bacteria, media, and growth conditions

Liquid overnight and solid cultures of *S. pyogenes* 5448 strain (*emm* type 1, M1T1) (69), SP79 (*emm* type 28, M28), and the clinical isolates CI407 (*emm* type 90, M90) and CI543 (*emm* type 1, M1) (70) were grown aerobically at 37°C in Todd-Hewitt broth supplemented with 2% yeast extract (BD, France). Liquid and solid cultures of *L. plantarum* and *L. rhamnosus* were grown aerobically at 37°C in De Man Rogosa and Sharpe medium (Merck, Germany). *Staphylococcus aureus* strain USA 300 JE2 was obtained from the EBI NARSA collection (NR-46543). Liquid growth of *S. aureus* USA 300 JE2 was done aerobically at 37°C in Tryptic soy broth (TSB; BD, France), while solid growth was done in Columbia-blood agar (Biomerieux).

### Bacteria reconstitution, isolation, and identification

Lyophilized *L. plantarum* Lp 115 SD-5209 and *L. rhamnosus* GG SD-7017 are marketed by Renew Life (UK) in Florabiotic Everyday Plus capsules. Maximum recovery diluent (Oxoid, UK) was used as a diluent for bacterial reconstitution. Reconstituted bacteria were diluted and plated on MRS and incubated aerobically at 37°C (71). Individual colonies were acquired randomly and spotted onto MALDI-TOF Biotyper target plates. A volume of 1 µL of MALDI matrix (10 mg/mL solution of α-cyano-4-hydroxycinnamic acid in 50% acetonitrile/2.5% trifluoroacetic acid) was added onto each spot and dried. MALDI-TOF/Microflex LT (Bruker Daltonics, USA) was used for automatic measurement and data interpretation. Isolates with logs (scores) ≥ 2 were accepted as correct species identification. Accepted colonies were replated and incubated aerobically at 37°C before reconfirmation with MALDI-TOF following the same procedure (72). Whole-genome sequencing was performed for both isolated strains to confirm species identity.

### Cell-free supernatant preparation

*L. plantarum* and *L. rhamnosus* were cultured aerobically at 37°C for 24 hours in MRS broth, THY broth, or Roswell Park Memorial Institute 1640 (Gibco) supplemented with 3% THY. *S. pyogenes* was grown for 24 hours in THY. *S. aureus* was grown for 24 hours in TSB. Bacterial suspensions were centrifuged (3,350 *g* for 10 minutes), and supernatants were filtered with 0.22 µm filters (TPP, Switzerland). Cell-free supernatants of *L. plantarum* and *L. rhamnosus* were stored at 4°C until further use (52). To obtain *S. pyogenes* cell-free spent medium (GASSN) or *S. aureus* cell-free spent medium (SASN), *S. pyogenes* or *S. aureus* cell-free spent medium pH was adjusted to the same pH as the MRS cell-free supernatants LPSN and LRSN using 12 M HCL to a final pH of 4.5.

### Determination of lactate concentration

Lactic acid concentrations were assessed in *L. plantarum* and *L. rhamnosus* supernatants grown in either MRS, THY, or RPMI supplemented with 3% THY for 24 hours. Cell-free supernatants were collected following the previously described procedure. Lactate was quantified using a lactate assay kit (D-lactic/L-lactic acid UV method, r-biopharm, Roche). All samples were measured using fresh growth medium as a control.

### *S. pyogenes* growth determination and liquid culture inhibition assay

*S. pyogenes* and *S. aureus* liquid cultures were prepared by dilution of an overnight culture to OD_600_ of 0.1 in RPMI (Gibco) supplemented with 3% THY or plain RPMI, respectively. Growth was done in the presence or absence of 5% LPSN and LRSN. The LPSN and LRSN were prepared in THY, RPMI, or MRS, with MRS supernatants chosen for all the biofilm assays. Overnight cultures were washed once with sterile PBS before dilution. Survival rates were determined by CFU/mL counts every hour (72). RPMI with 3% THY was used as a control for *S. pyogenes* growth.

### Biofilm determination by crystal violet staining

Biofilms were washed with PBS at the end of the incubation times (24, 48, and 72 hours). The liquid was removed, and the biofilm was dried for 1 hour at 50°C. Next, a solution of 0.1% crystal violet was added (1:10 diluted in MilliQ H_2_O), and the biofilms were incubated for 30 minutes at RT after which the biofilms were washed with MilliQ H_2_O. The liquid was removed, and the stained biofilm was dissolved in 90% EtOH. An aliquot of the solution was taken into a 96-well plate, and the absorbance was measured at 570 nm.

### *S. pyogenes* biofilm viability assay

*S. pyogenes* overnight cultures were washed with sterile PBS once before diluting to OD_600_ of 0.1 in RPMI (Gibco) supplemented with 3% THY. Two-milliliter aliquots were transferred to 6-well tissue culture plates (TPP, Switzerland) and incubated aerobically at 37°C. LPSN or LRSN (1%, 5%, 10%, or 20% concentrations) obtained from growth in MRS were added after 24 hours of incubation to the pre-formed *S. pyogenes* biofilms. For biofilms grown with medium exchange every 24 hours, LPSN or LRSN were freshly added along with the medium change. Twenty percent GASSN was used as a control for the effect of spent medium and low pH, while 20% SASN was used as an additional treatment to compare against the lactobacillus supernatant. After 72 hours of incubation, the supernatant of each well was collected, biofilms were washed three times with sterile PBS, and attached bacterial cells were removed mechanically by sonication and scratching the bottom of the wells using a 200 µL micropipette tip until the bottom of the well was visibly clear. Supernatants and biofilm suspensions were serially diluted, plated on THY agar, and incubated aerobically at 37°C for CFUs’ enumeration. Viable counts were expressed as the CFU per mL.

### Treatment of cell-free supernatants

In order to assess the origin of the bioactive compound from LPSN and LRSN grown in MRS, an enzymatic treatment was performed. α-amylase (150 units/mL, Sigma-Aldrich), proteinase K (1 mg/mL, Sigma-Aldrich), and lipase (25 mg/mL, Sigma-Aldrich) were used. The different enzymes were added individually or in combination with both LPSN and LRSN and incubated at 37°C for 3 hours, followed by heat inactivation at 95°C for 3 minutes. The samples were centrifuged (3,350 × *g* for 3 minutes), and the supernatant was stored at 4°C until further use (72). Double enzymatic treatments were performed in two steps, as described above. For all conditions, enzyme-treated GASSN was used as a control. Adjustment of lactic acid concentration in cell-free supernatants was performed by dilution of a 2 M L-lactate stock (Sigma-Aldrich, Belgium) to 140 mM in GASSN.

### Visualization of *S. pyogenes* biofilms

*S. pyogenes* overnight cultures were washed with sterile PBS once before diluting to OD_600_ of 0.1 in RPMI supplemented with 3% THY. Aliquots were transferred to 8-well µ-slides (Ibidi GmbH, Germany) and incubated aerobically at 37°C. MRS LPSN and LRSN were added (20%, vol/vol) at 0 hour or after 24 hours of *S. pyogenes* incubation. Growth medium containing MRS LPSN or LRSN derived was exchanged every 24 hours. GASSN was used as a control. After 72 hours, biofilms were washed three times with sterile PBS, fixed at 50°C for 50 minutes, and stained with LIVE/DEAD BacLight Kit (Molecular Probes-Invitrogen, USA). Biofilms were visualized by confocal laser scanning microscopy (CLSM-Leica SP8 inverse, Center for Microscopy, University of Zurich). Biofilm volume (µm^3^) and mean intensity of red channel (dead or damaged bacteria stained by PI) values of three different biological replicates and two technical replicates for each condition were retrieved using the Imaris Microscopy Image Analysis Software (Oxford Instruments, UK).

### Characterization of *L. plantarum* and *L. rhamnosus* cell-free supernatants by Fourier transmission infrared spectroscopy

Cell-free supernatants of *L. plantarum* and *L. rhamnosus* grown in MRS and in supplemented RPMI were obtained as described previously. Functional groups present in the supernatants were analyzed using FTIR. FTIR spectra for both cell-free supernatants and fresh medium were recorded in the region of 400–4,000 cm^−1^ at a resolution of 4 cm^−1^ on an ALPHA FTIR Routine spectrometer (Bruker) (73). Four independent replicates for each condition were analyzed. Functional group data analysis was performed on KnowItAll System 2021–Spectroscopy Edition (John Wiley & Sons, Inc.).

### Statistical analysis

Statistical analysis of the data was performed with the software Graphpad Prism (version 9.5.1), and the different tests were performed after normality was assessed using the Shapiro-Wilk test and QQ plots. Growth curves were analyzed using a non-linear regression with either Gompertz growth or beta growth then decay models, followed by a one-way ANOVA. Normally distributed data were analyzed by ordinary one-way ANOVA, while for non-parametric data, Kruskal-Wallis or a mixed-model analysis was used. All tests were further evaluated *post hoc* for multiple comparisons using Benjamini, Krieger, and Yekutieli correction for false discovery rate. A significance threshold of *P* < 0.05 or *q* < 0.05 was considered for all experiments.

## SUPPLEMENTAL MATERIAL

Supplemental figures msphere.00430-24-s0001.docx Figures S1 to S7.

ASM does not own the copyrights to Supplemental Material that may be linked to, or accessed through, an article. The authors have granted ASM a non-exclusive, world-wide license to publish the Supplemental Material files. Please contact the corresponding author directly for reuse.
