# Supplementary material for: Inhibition of Streptococcus pyogenes biofilm by Lactiplantibacillus plantarum and Lacticaseibacillus rhamnosus
Source: mSphere. 2024 Oct 3;9(10):e00430-24. doi: 10.1128/msphere.00430-24 (PMC11520294; doi:10.1128/msphere.00430-24)
Supplement: Supplemental figures — Figures S1 to S7. [file msphere.00430-24-s0001.docx]

**Inhibition of *Streptococcus pyogenes* biofilm by *Lactiplantibacillus plantarum* and *Lacticaseibacillus rhamnosus***

Alejandro Gómez-Mejia^1*^; Mariano Orlietti^1*^; Andrea Tarnutzer^1^; Srikanth Mairpady Shambat^1^; Annelies S. Zinkernagel^1+^

1 Department of Infectious Diseases and Hospital Epidemiology, University Hospital Zurich, University Zurich, Switzerland

**Running title: Group A *Streptococcus* biofilm inhibition by *Lactibacillus***

+ Address correspondence to Annelies S. Zinkernagel, Annelies.Zinkernagel@usz.ch

* Alejandro Gómez-Mejia and Mariano Orlietti contributed equally to this work. Author order was determined based on seniority.

**Supplementary material**

**
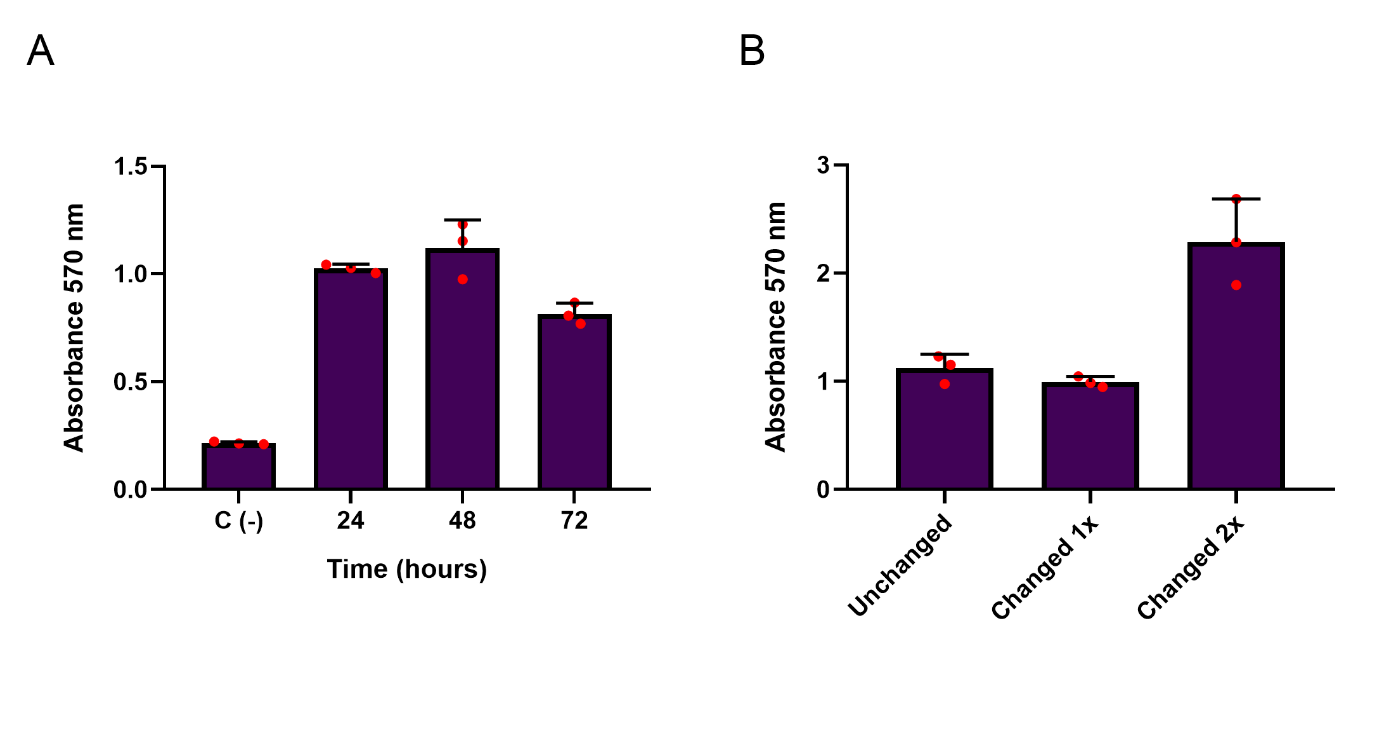
Figure S1**. Crystal violet measurements of *S. pyogenes* biofilms in RPMI supplemented with 3% THY. (**A**) OD_570_ measurements of crystal violet staining of *S. pyogenes* biofilms after 24, 48 and 72 hours of static growth without medium exchange. (**B**) OD_570_ measurements of crystal violet staining of *S. pyogenes* biofilms after 72 hours of growth without medium exchange, medium exchange once after 24 hours and medium exchange every 24 hours. Background signal from the crystal violet is shown as C (-). The experiments were performed in biological triplicates.


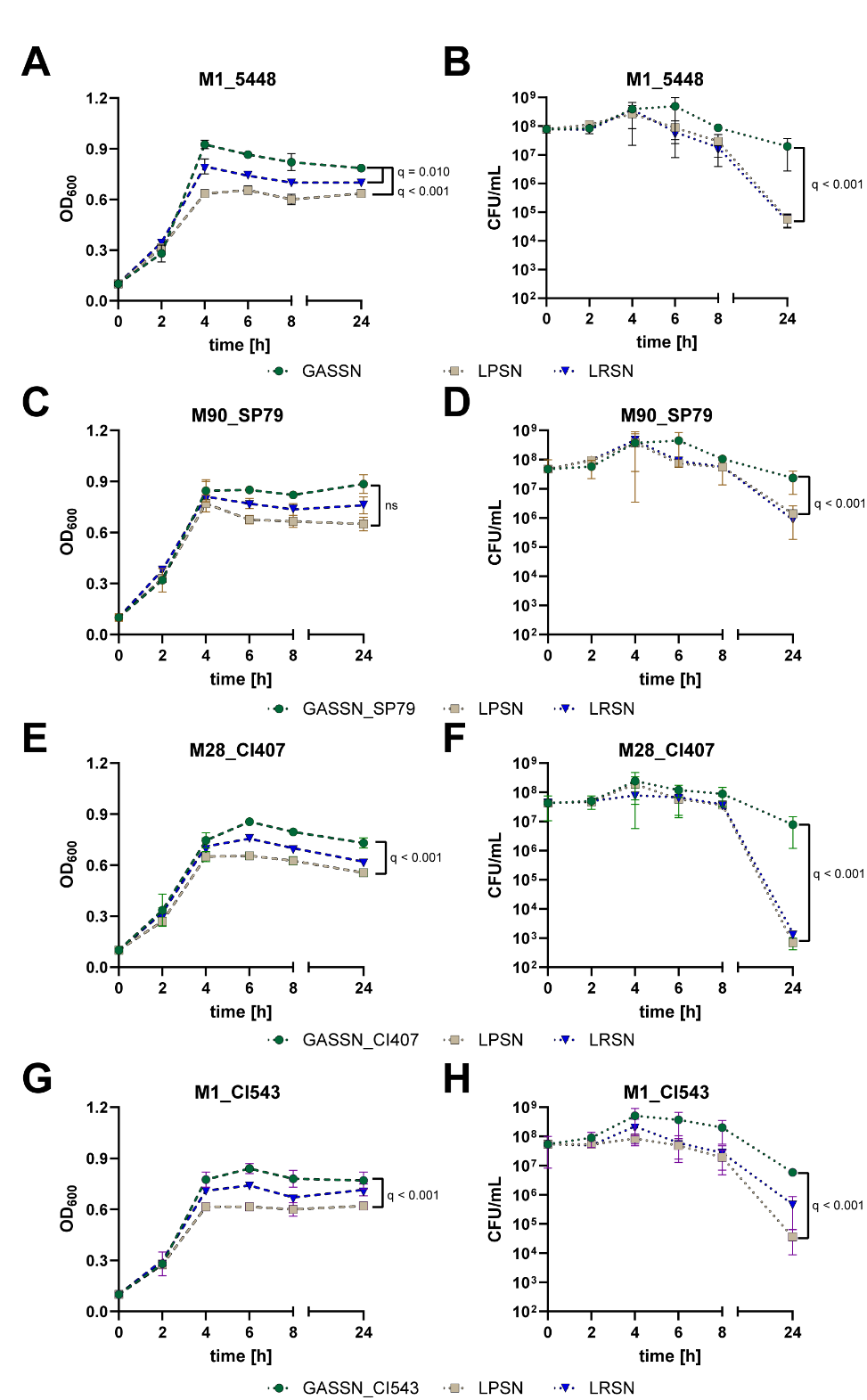


**Figure S2.** **Cell free supernatant from L. plantarum (LP) and L. rhamnosus (LR) inhibits the growth of different emm-type S. pyogenes strains.** Determination of the inhibitory effect of 5 % v/v LPSN or LRSN on the growth of 4 different S. pyogenes strains measured by optical density (OD_600_) (**A, C, E and G**) or as CFU/mL (**B, D, F, H**). As a control, GASSN treatment was used consisting of cell-free spent medium adjusted to pH 4.5 from each different S. pyogenes strain. Data shown as mean ± SD of three independent replicates (n=3). Statistical significance is indicated as q-values. The statistic test used were a non-linear regression model followed by a one-way ANOVA analysis of the growth rate (**OD)** or a one-way ANOVA analysis of the population at peak (CFU/mL). All cases were analysed with correction for false discovery with Benjamini, Krieger and Yekutieli multiple comparison.

**
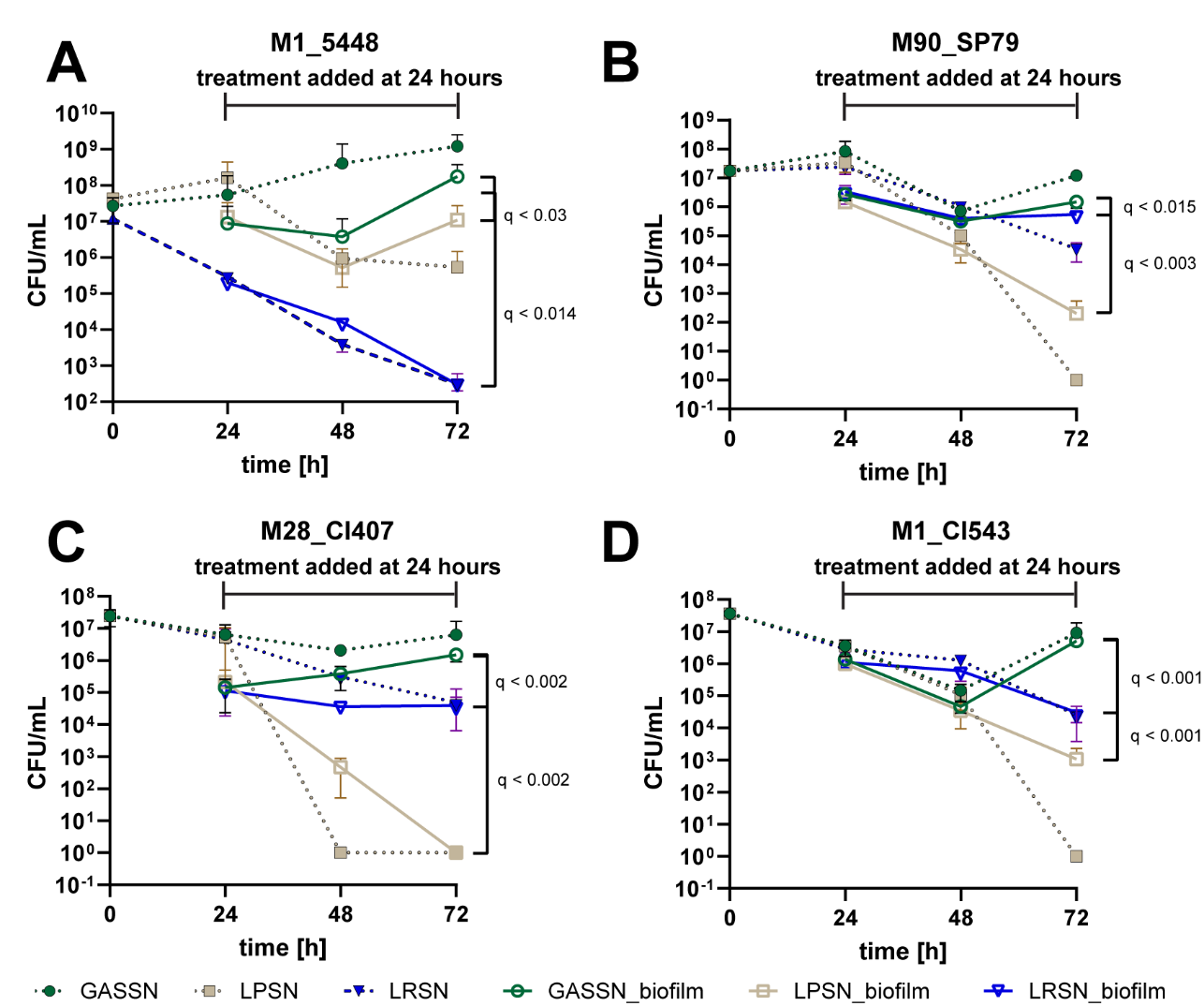
**

**Figure S3.** **Cell free supernatant from *L. plantarum* (LP) and *L. rhamnosus* (LR) significantly reduces biofilms formed by different *emm*-type *S. pyogenes* strains.** Determination of the inhibitory effect of 20 % v/v LPSN or LRSN on 24 hours-formed biofilm from four different *S. pyogenes* strains (5448, SP79, CI407 and CI543) measured as viable CFU/mL. As a control, GASSN treatment was used consisting of cell-free spent medium adjusted to pH 4.5 from each different *S. pyogenes* strain. Treatment was added after 24 hours of biofilm formation. Medium with treatment was exchanged every 24 hours. Data shown as mean ± SD of three independent replicates (n=3). Statistical significance is indicated as q-values. The statistic test used was an ordinary one-way ANOVA followed with correction for false discovery with Benjamini, Krieger and Yekutieli multiple comparison

*
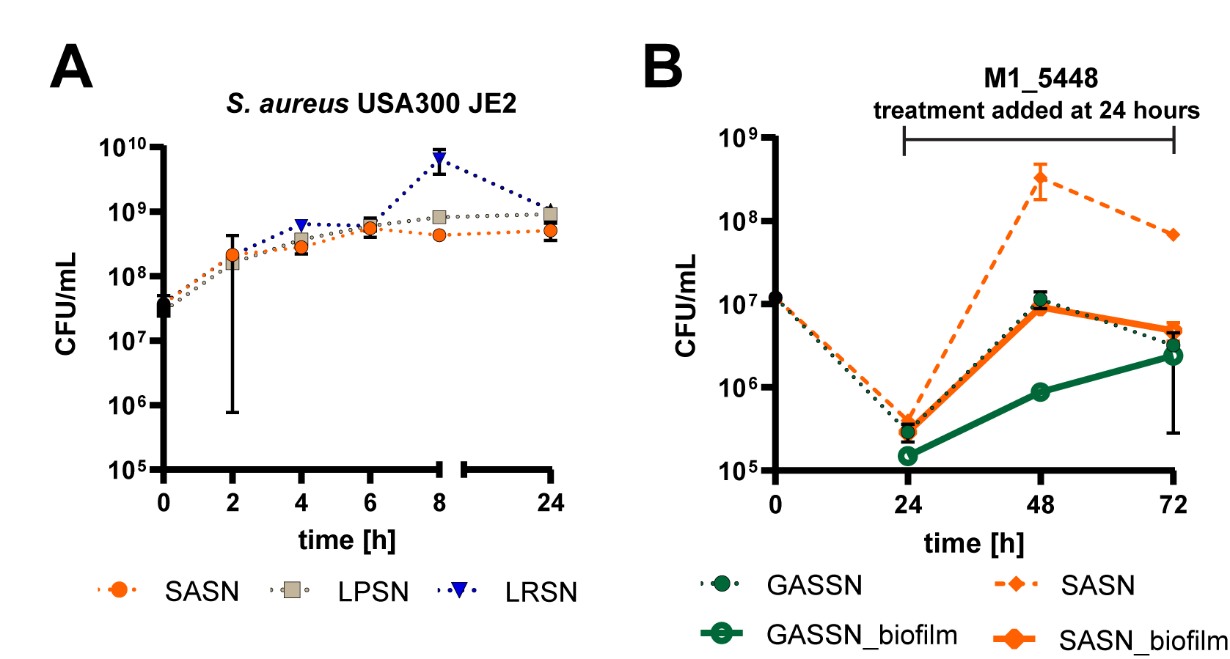
*

**Figure S4.** **Effect of LP and LR cell free supernatant on S. aureus USA300 JE2 viability.** Determination of the inhibitory effect of 5 % v/v LPSN or LRSN on the growth of S. aureus USA300 JE2 measured as CFU/mL. As a control, SASN (S. aureus USA300 JE2 cell-free spent TSB medium adjusted to pH 4.5) treatment was used (**A**). Assessment of the inhibitory effect of 20 % v/v SASN on 24 hours-formed biofilm of S. pyogenes strain 5448 measured as viable CFU/mL As a control, GASSN treatment was used consisting of cell-free spent medium adjusted to pH 4.5 from S. pyogenes 5448 strain, Treatment was added after 24 hours of biofilm formation. Media with treatment was exchanged every 24 hours. Data shown as mean ± SD of three independent replicates (n=3). No statistical significance was found in any of the conditions. The statistic test used were a non-linear regression model followed by a one-way ANOVA analysis of the population at peak and an ordinary one-way ANOVA. All cases were analysed with correction for false discovery with Benjamini, Krieger and Yekutieli multiple comparison.


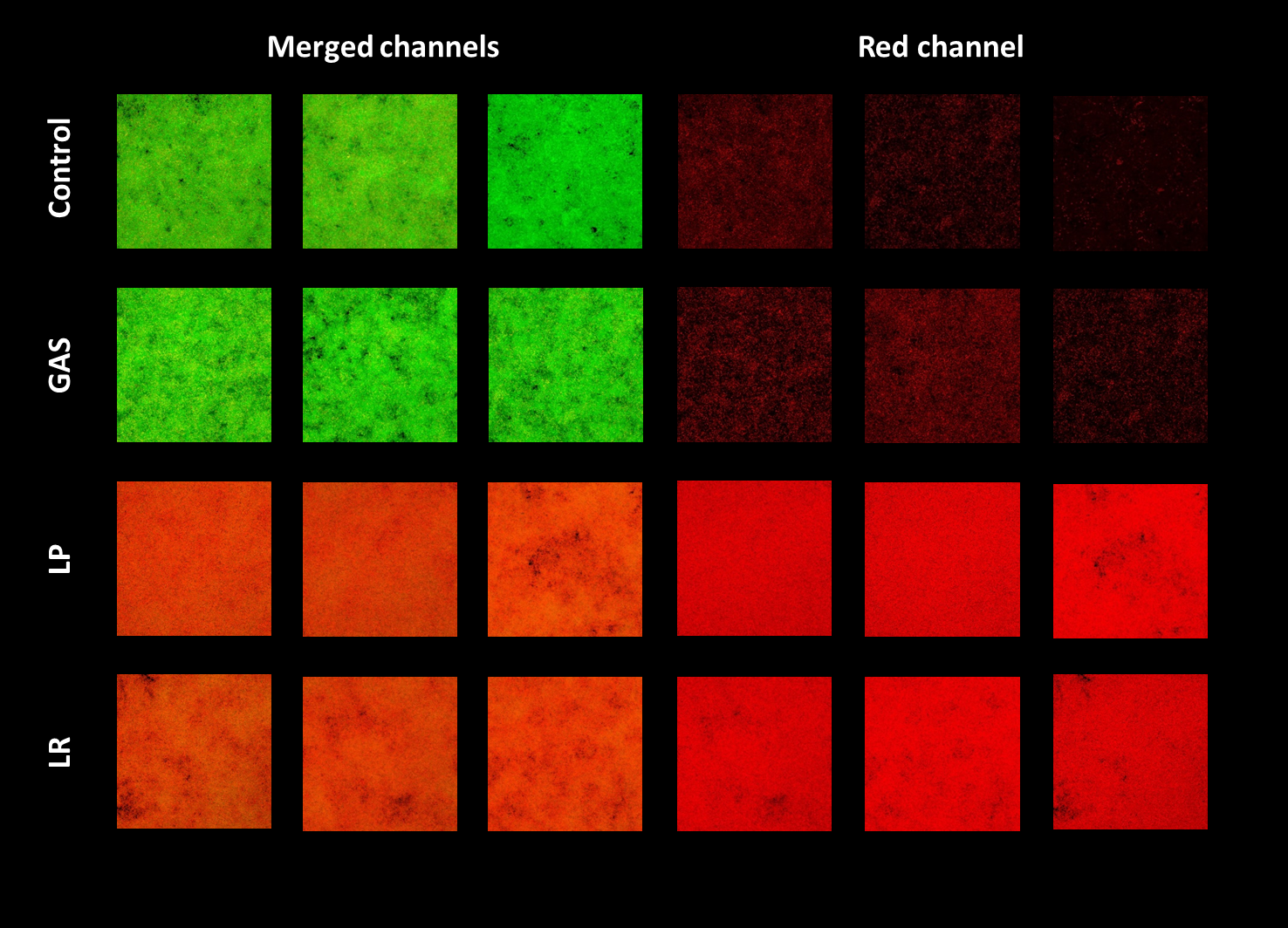


**Figure S5**. 2D models of 72 hours S. pyogenes biofilms (n= 3) treated with 20% v/v of MRS LPSN or LRSN at hour 0. “Control” = untreated S. pyogenes biofilms. “GAS” = S. pyogenes biofilms treated with 20% GASSN. ”LP” = S. pyogenes biofilms treated with 20% LPSN. “LR” = S. pyogenes biofilms treated with 20% LRSN. Biofilms are stained using Syto9 (green channel, live bacteria) and propidium iodide (red channel, dead or membrane damaged bacteria) and imaged with a confocal laser scanning microscope.


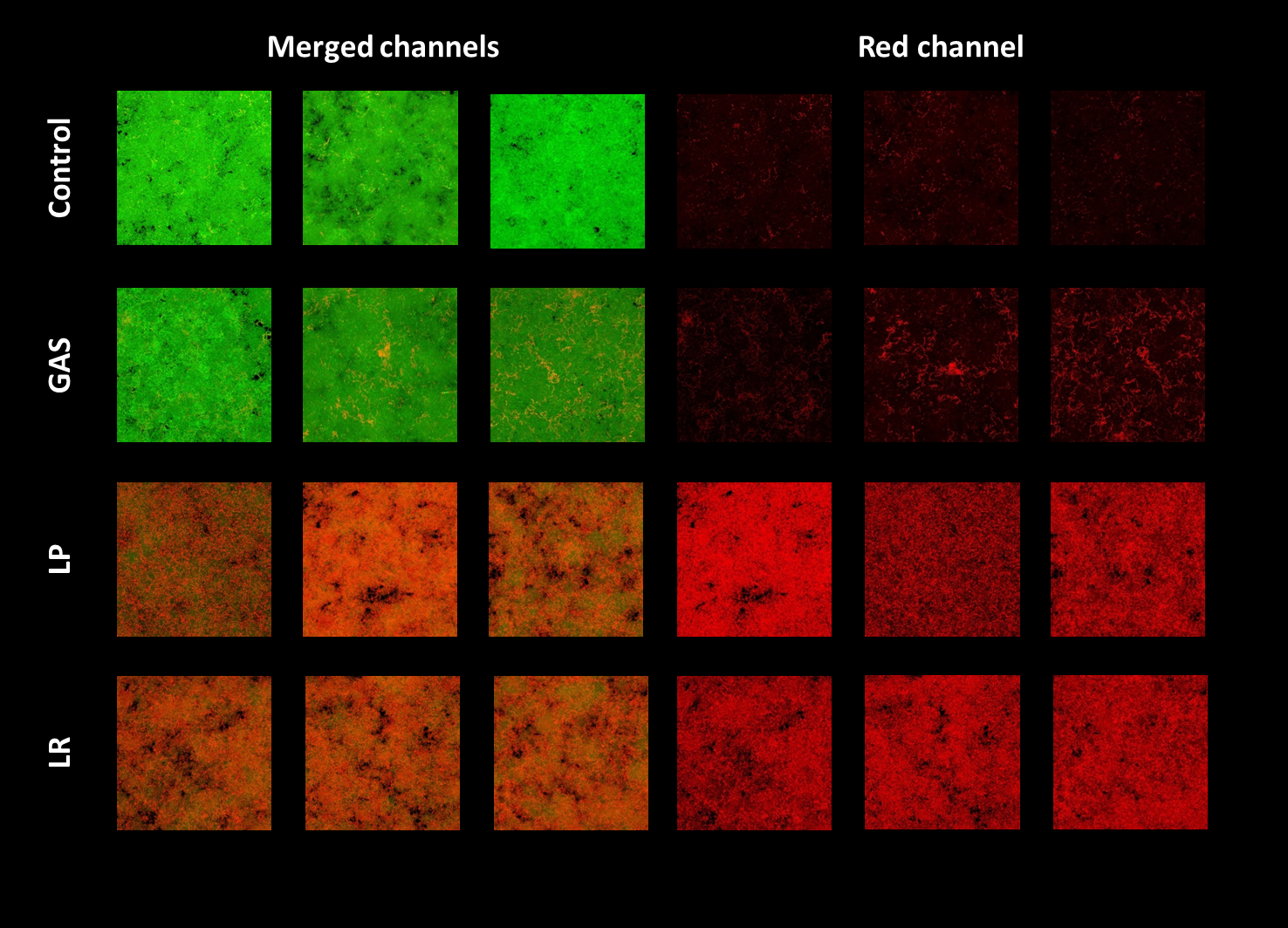


**Figure S6.** 2D models of 72 hours S. pyogenes biofilms (n = 3) treated with 20% v/v of MRS LPSN or LRSN after 24 hours of biofilm formation. “Control” = untreated S. pyogenes biofilms, “GAS” = S. pyogenes biofilms treated with 20% GASSN, “LP” = S. pyogenes biofilms treated with 20% LPSN, “LR” = S. pyogenes biofilms treated with 20% LRSN. Biofilms are stained using Syto9 (green channel, live bacteria) and propidium iodide (red channel, dead or membrane damaged bacteria) and imaged with a confocal laser scanning microscope.
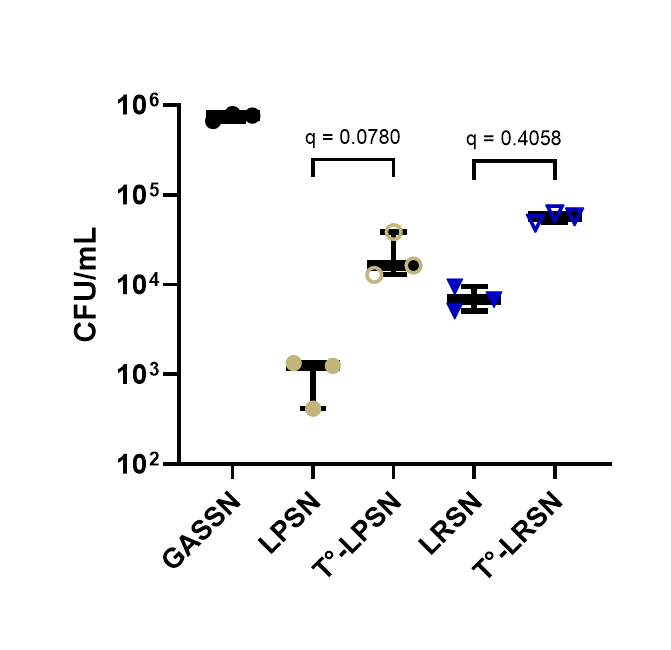


**Figure S7.** Effect of exposure to 95°C for three minutes on the antibacterial activity of 20% MRS LPSN and LRSN (T°-LP and T°-LR). The prepared supernatants were added to biofilms after 24 hours of growth in culture media supplemented with THY and incubated for a further 48 hours (72 hours total incubation time with media exchange every 24 hours). The CFUs were enumerated for all conditions. Data is shown as mean ± SD of three independent replicates (n=3). Statistical significance is indicated as q-values. The statistic test used was an ordinary one-way ANOVA with Benjamini, Krieger and Yekutieli multiple comparison correction for false discovery rate.
